# The Relationship Between Short-Chain Fatty Acid Secretion and Polymorphisms rs3894326 and rs778986 of the *FUT3* Gene in Patients with Multiple Sclerosis—An Exploratory Analysis

**DOI:** 10.3390/nu18010062

**Published:** 2025-12-24

**Authors:** Monika Kulaszyńska, Wiktoria Czarnecka, Natalia Jakubiak, Daniel Styburski, Mateusz Sowiński, Norbert Czapla, Ewa Stachowska, Dorota Koziarska, Karolina Skonieczna-Żydecka

**Affiliations:** 1 Department of Biochemical Science, Pomeranian Medical University in Szczecin, Broniewskiego 24, 71-460 Szczecin, Poland; monika.kulaszynska@pum.edu.pl (M.K.);; 2 Department of Human Nutrition and Metabolomics, Pomeranian Medical University in Szczecin, Broniewskiego 24, 71-460 Szczecin, Poland; 3 Sanprobi sp. z o.o. sp.k., Kurza Stopka 5/c, 70-535 Szczecin, Poland; 4 Clinic of Plastic, Endocrine and General Surgery, Pomeranian Medical University in Szczecin, Independent Public Clinical Hospital No. 1, ul. Siedlecka 2, 72-010 Police, Poland; 5 Department of Neurology, Pomeranian Medical University in Szczecin, Unii Lubelskiej 1, 72-252 Szczecin, Poland

**Keywords:** *FUT3*, multiple sclerosis, neurodegeneration, polymorphism

## Abstract

**Background:** The intestinal microflora is a population of microorganisms that resides in the human gastrointestinal tract and is important in maintaining metabolic and immune homeostasis in the body. Bacteria residing in the intestine produce short-chain fatty acids (SCFAs), which communicate with, among other things, the brain–gut axis—disorders of which are one of the causes of MS-like pathologies. A particular property of SCFAs is the induction of regulatory T cells, which are finding their way into pioneering therapies for MS patients. The aim of the study is to evaluate SCFA secretion in patients with multiple sclerosis from the West Pomeranian region depending on the genotypes of rs778986 and rs3894326 polymorphisms of the *FUT3* gene. **Methods:** The study group included 47 patients clinically diagnosed with MS. Genotyping was performed by real-time PCR using TaqMan probes. Analysis of short-chain fatty acids in faeces was performed on a quadrupole mass spectrometer coupled to a time-of-flight (QTOF) analyser coupled to an AB Sciex high-performance liquid chromatograph (UHPLC). **Results:** Statistical analysis did not reveal any statistically significant differences in the prevalence of the studied polymorphisms in MS patients compared to the healthy control group. It was observed that the intestinal microflora and SCFA production in MS patients may be disturbed, while the studied *FUT3* gene polymorphisms probably do not have a significant effect on their concentrations. A statistical tendency towards higher caproic acid content in heterozygotes of the rs778986 polymorphism and higher valeric acid secretion in homozygotes of rs3894326 was demonstrated. **Conclusions:** In summary, the studied *FUT3* gene polymorphisms are not overrepresented in patients with MS. The rs778986 *FUT3* polymorphism may affect the caproic acid content in the faeces of patients with MS, and the rs3894326 polymorphism may affect valeric acid secretion. Due to the small sample size and sparse genotype groups, the study has limited power and negative findings may reflect Type II error; replication in larger cohorts is warranted.

## 1. Introduction

The brain–gut axis is a complex communication system connecting the nervous and digestive systems, whose interactions play an important role in regulating physiological processes. An increasing number of studies focus on microbial endocrinology, which assumes the existence of common neurotransmitters for the host and microbiota. It has been proven that intestinal bacteria can synthesise neuroactive compounds that affect the human body through the brain–gut axis, such as catecholamines, tryptophan, and serotonin [1]. The complex links between the nervous and digestive systems point to a possible connection between the gut microbiota and the development of many neurological and autoimmune diseases, including MS. A disturbed microbiota can also modify the innate and adaptive immune response by regulating pro- and anti-inflammatory cytokines [2].

The ratio of commensal to pathogenic bacteria is crucial for maintaining microbial balance, as commensals are responsible, among other things, for the synthesis of short-chain fatty acids (SCFAs). These compounds play a multifaceted role in maintaining intestinal health: they support the integrity of the intestinal barrier, reduce the risk of developing colon cancer, stimulate mucus production, and modulate inflammation by influencing cytokine secretion. SCFAs are key mediators in the communication between the microbiota and the immune system, enabling the maintenance of a balance between pro- and anti-inflammatory processes [3]. The most important of these are acetate, propionate, and butyrate, usually occurring in a molar ratio of 3:1:1. However, these proportions may change under the influence of environmental factors such as diet, age and lifestyle [4]. The largest producers of butyrate are bacteria from the Lachnospiraceae and Ruminococcaceae families, the main producers of propionate are Bacteroidetes, Negativicutes, and Lachnospiraceae, and the main producer of acetate is Bifidobacteriaceae [5].

**Figure 1:**
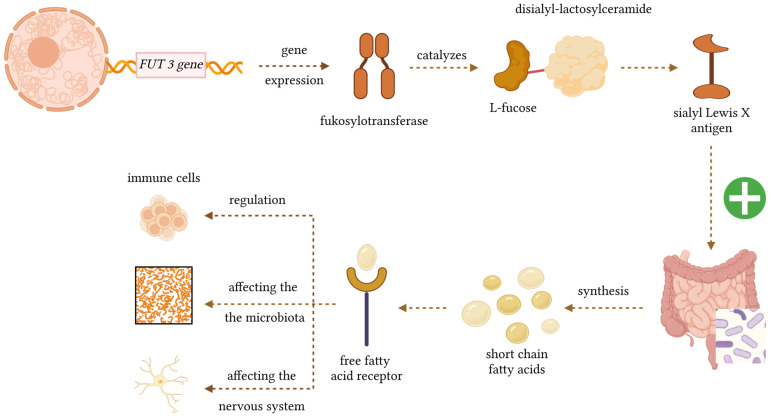
The influence of the *FUT3* gene on the gut microbiota and the production of SCFAs affecting the body’s cells. Created in Biorender. Wiktoria Czarnecka. (2025) https://BioRender.com/bon6ve0.

An important factor in regulating the gut microbiota is lifestyle, but genetic factors also play a role. The *FUT3* gene, located on the short arm of chromosome 19, encodes the enzyme alpha-1-3-fucosyltransferase (Lewis enzyme), which catalyses the transfer of the L-fructose residue from GDP-fructose to the appropriate sugar residues of polysaccharide precursors. *FUT3* expression is particularly high in many epithelial tissues: the stomach, intestine, lungs, kidneys, and to a lesser extent in the salivary glands, bladder, uterus, and liver. In cooperation with the *FUT2* gene product, it participates in the formation of Lewis blood group antigens [6]. These antigens are produced outside erythrocytes and are then adsorbed onto glycosphingolipids in cell membranes and tissues such as the intestines, pancreas, and stomach. There are six Lewis system antigens, Lea, Leb, Leab, LebH, ALeb, and BLeb, of which Lea and Leb are mainly clinically significant. They appear in children around 6 months of age [7]. The *FUT3* gene also determines the formation of an important surface antigen—Sialyl Lewis X (sLeX)—which is a tetrasaccharide enabling leukocyte rolling and adhesion to the vascular endothelium and their migration to target tissues through interaction with selectins [8]. Selectins (especially E-selectin) and their ligands (e.g., sLeX) mediate the passage of lymphocytes through the endothelium into the brain and spinal cord—a mechanism by which autoreactive T cells can enter the CNS and cause demyelination. This directly links the function of sLeX to the pathogenesis of MS. Liu et al. indicate that targeting 6-sulfo-sLeX prevents the progression of EAE (experimental autoimmune encephalomyelitis, an animal model of MS). This suggests that inhibiting the function of these glycans may have modulatory and therapeutic potential in MS [9]. Mechanistic Figure 1 shows a biological diagram illustrating how the *FUT3* gene influences SCFA production via the gut microbiota and how SCFAs interact with various cell types in the body.

The importance of the *FUT3* gene in the human body begins as early as the prenatal period, and the first years of life are crucial for the proper development of the gut microbiota. It is believed that genes encoding fucosyltransferases, including *FUT3*, play an important role in shaping the microbiota [10]. Individuals with an active *FUT3* gene exhibit the presence of numerous sialic antigens on the surface of cells, which resemble sialic acids. Commensal bacteria, like many gastrointestinal pathogens, are capable of metabolising sialic acids, which determines the phenomenon of competition between them [11]. The regulation of *FUT3* expression is not limited to genotype alone; as a review of the literature shows, the transcription and translation of *FUT3* is controlled by many factors: transcription factors, RNA-binding proteins, and microRNAs, which influence the actual expression of the enzyme in epithelial cells [12]. Fucosyltransferase enzymes also participate in the synthesis of human milk oligosaccharides (HMOs) [13]. Approximately 200 different HMOs resistant to digestion in the upper intestine provide a source of nutrition for intestinal microorganisms, while also performing a protective function by blocking the adhesion of pathogens to the intestinal epithelium and preventing infections. Bacteria unable to attach to the epithelium are eliminated without damaging the tissue, minimising the risk of toxic metabolite production. *FUT3* gene polymorphisms may influence the fucosylation process and thus shape resistance or susceptibility to certain diseases.

Consequently, the major correlates between MS and *FUT3* gene include the role of *FUT3* in regulating the biosynthesis of Lewis antigens, thereby shaping host–microbiota interactions. This matters because fucosylated glycans can influence microbial colonisation and mucosal communication, and the gut microbiota is increasingly recognised as a factor affecting both MS risk and clinical course [14,15]. Also, *FUT3* gene polymorphisms (including rs778986 and rs3894326) might affect SCFA secretion [11], and these might also play a role in the MS pathophysiology components (Figure 1) [16]. Secondly, *FUT3*-dependent glycan fucosylation patterns modulate immune responses. This is relevant because disruptions in fucosylation have been associated with autoimmune diseases and may contribute to, or modify, immune dysregulation central to MS immunopathology [17,18]. Also, the influence of *FUT3* on fucosylation pathways implicated in neuroinflammatory signalling. This is important because experimental evidence suggests that altered fucosylation can affect CNS inflammation and demyelination—core features of MS [19,20,21].

In view of the above, the main objective of this study was to evaluate SCFA secretion in patients with multiple sclerosis from the West Pomeranian region depending on the genotypes of rs778986 and rs3894326 polymorphisms of the *FUT3* gene. Given that changes in *FUT3* expression/activity modify the composition and structure of intestinal mucosal glycans, changes in glycan patterns select a different pool of species, e.g., reduce the number of those that produce SCFA, and that reduced concentrations of certain SCFA have been reported in MS patients, a hypothesis has been put forward that the studied *FUT3* gene polymorphisms may negatively affect SCFA secretion in patients with multiple sclerosis.

## 2. Materials and Methods

### 2.1. The Study Group

The study group consisted of 47 patients (32 women and 15 men) with a clinical diagnosis of MS, under the care of the Provincial Centre for Demyelinating Diseases in Szczecin. The patients’ disability was assessed by two neurologists at the time of diagnosis and in 2019 using the Expanded Disability Status Scale (EDSS). The study was conducted in accordance with the Declaration of Helsinki and was approved by the Bioethics Committee of the Pomeranian Medical University in Szczecin (approval no. KB-0012/163/12). All eligible participants provided written informed consent prior to inclusion. No control group was recruited. When comparing the results of genotyping we used data reported in the scientific database (1000 Genomes).

### 2.2. DNA Isolation

Genomic DNA was isolated from peripheral blood leukocytes using the ExtractMe DNA Blood Kit (BLIRT, Gdańsk, Poland). The procedure was conducted in accordance with the manufacturer’s recommendations.

### 2.3. Identification of the Studied Polymorphisms

Genotyping was performed using real-time PCR with a Cycler^®^ 96 System (Roche Diagnostics, Pleasanton, CA, USA) using TaqMan probes (Life Technologies, Foster City, CA, USA). Excited signals were detected using FAM and VIC fluorescent dyes.

The reaction mixture (10 μL) contained:

- 1 μL genomic DNA;
- 5 μL TaqMan Genotyping Master Mix (Life Technologies, Foster City, CA, USA);
- 3.75 μL PCR Grade Water (Life Technologies, Foster City, CA, USA);
- 0.25 μL TaqMan probe (Life Technologies, Foster City, CA, USA).

Real Time PCR was performed under the following conditions:

- Pre-incubation (1 cycle): 300 s—95 °C.
- 2-step amplification (50 cycles):
  1. 95 °C × 15 s;
  2. 60 °C × 60 s.

The cooling stage was omitted. The 1000 genomes database was used to determine whether the frequencies of genotypes and alleles of the studied polymorphism in our study group differ significantly in relation to the healthy population.

### 2.4. SCFA Isolation

Short-chain fatty acids were isolated from patient stool samples using the Zhao method with Roediger’s modification. In total, 0.5 g of stool sample was dissolved in 5 mL of water and homogenised for 5 min. A 5 molar HCl solution was then added to lower the pH to 2–3, followed by shaking for 10 min and centrifugation for 20 min at 5000 rpm. A clear supernatant was obtained, which was filtered through a membrane with a filtration diameter of 400 µm. It was transferred to a chromatography vial.

The collected material was analysed to assess the concentration of the following short-chain fatty acids:

- Acetic acid C2;
- Propionic acid C3;
- Butyric acid C4;
- Valeric acid C5;
- Caproic acid C6.

### 2.5. SCFA Analysis

SCFA analysis in faeces was performed using a quadrupole mass spectrometer coupled with a time-of-flight analyser (QTOF) connected to a high-performance liquid chromatograph (UHPLC) from AB Sciex—TripleTOF^®^ 6600+ Marlborough, MA, USA. Freshly thawed faecal samples (60 mg wet weight) were dissolved in 300 µL MeOH, vortexed at 1600 rpm for 5 min, treated with ultrasound for 30 min at room temperature, and vortexed again. After centrifugation (18,000× *g* for 15 min at 4 °C), the supernatants were transferred and stored at −80 °C until analysis. Prior to transfer to chromatography vials, all samples were centrifuged at 18,000× *g* for 30 min at 4 °C. The samples were analysed using the TripleTof^®^ 6600+ system Marlborough, MA, USA. Compounds were separated on an Acquity UPLC HSS T3 (C18) column (1.8 µm, 2.1 × 100 mm, Waters, Wexford, Ireland) at 45 °C. The injection volume was 2 µL. Separation was performed using a gradient of buffers—water (A) and methanol (B)—both containing 0.04% formic acid, which transported the sample through the column at a flow rate of 0.4 mL/min. Gradient schedule—0–6 min: 5%–100% B; 6–10.50 min: 100% B; 10.50–10.51 min: 100% to 5% B; 10.51–15 min: 5% B. Ionisation was performed using electrospray ionisation (ESI) San Diego, CA, USA at a voltage of 3500 V in positive and negative modes, using nitrogen as the nebulising and drying gas. Gas temperature at the source—175 °C, gas flow 12 L/min. Data analysis was performed using the metabolomic analysis system XCMSplus San Diego, CA, USA. The results for individual acids are given in nM/mg of faeces.

The calibration curve was prepared by preparing 10 serial points of main stock dilutions with a specific concentration of the tested acids. QC samples and blank samples were also prepared. LOD was defined as a 10:1 signal-to-noise ratio:

- C2—0.82 nM/mg;
- C3—0.51 nM/mg;
- C4—0.26 nM/mg;
- C5—0.22 nM/mg;
- C6—0.17 nM/mg.

### 2.6. Statistical Analysis

Statistical analysis was performed using MedCalc^®^ (Statistical Software version 20.218). The distribution of continuous variables differed significantly from normal; therefore, descriptive statistics were presented as medians and interquartile ranges. Courts’ online calculator (2005–2008) was used to determine compliance with Hardy–Weinberg’s law. Differences in continuous variables depending on the genotype of the studied polymorphism were analysed using the Mann–Whitney U test or the Kruskal–Wallis test, while the Chi-square test was used to assess the relationships between qualitative data. A significance level of *p* < 0.05 was adopted, while *p* = 0.05–0.1 was considered to be an area of statistical tendency.

Because several genotype strata were rare in the present cohort, we anticipated limited power for codominant and recessive inheritance models, and therefore prioritised dominant and overdominant genotype contrasts to reduce sparse-cell bias and unstable estimates. In line with recommendations to avoid over-interpreting non-significant p-values as proof of no effect, null findings were interpreted primarily as absence of evidence rather than evidence of absence, unless accompanied by sufficiently narrow confidence intervals to exclude the effects of a magnitude considered biologically meaningful (smallest effect size of interest, SESOI).

## 3. Results

### 3.1. Characteristics of the Study Group

The study group consisted of 47 persons, with female predominance (n = 32; 68.1%), among whom the age of onset of the first symptoms was 28.6 years ± 7.7, ranging from 16 to 50 years. The average age of onset of the first symptoms is slightly higher in women (approximately 29.5 years) than in men (approximately 27.2 years). All subjects had relapsing-remitting multiple sclerosis (RRMS) and were undergoing immunomodulatory therapy. The mean EDSS score at the time of diagnosis was 1.50 points ± 0.88, while at the time of the study in 2019, it was 1.77 points ± 1.11. In practice, this score means that these are patients without mobility limitations and significant disease-related disability.

### 3.2. Genotyping

All biological samples tested were genotyped, but not all successfully. In the case of the *FUT3* gene polymorphism for rs778986 A>G, genotyping failed for 2 samples, while for rs3894326 T>A it failed for 1 sample. No significant deviation from Hardy–Weinberg equilibrium was observed in the genotype distribution, which was χ^2^ = 0.072, *p* = 0.79 for rs778986 A>G and χ^2^ = 2.45, *p* = 0.12 for rs3894326 T>A. In order to determine the potential relationship between the analysed polymorphisms and selected phenotypic traits, four classic allele inheritance models were used: codominant, dominant, overdominant, and recessive. Each of these models assumes a different way in which allele combinations influence a trait or disease risk, allowing for the identification of different mechanisms of genetic interaction.

The distribution of genotypes of the two studied *FUT3* gene polymorphisms (rs778986 and rs3894326) in various inheritance models (codominant, dominant, overdominant, and recessive) is presented in Table 1. The table is intended to assess the genetic structure of the sample and compare the models, and was used for further analysis presented in Table 2. For each variant, the number of observed genotypes and their percentage in the analysed group are presented. In the case of the rs778986 polymorphism, the highest frequency was found for the GG genotype (68.9%), while the AA genotype was rare (2.2%). Similarly, for the rs3894326 variant, the AA genotype dominated (87.0%), and the TT homozygote occurred sporadically (2.1%). In the dominant, overdominant, and recessive models, combined genotype categories were additionally presented, which allows for the assessment of potential allele dominance effects. Such a comparison allows for easy comparison of the observed genotype proportions between models and assessment of the potential nature of inheritance of the variants studied.

Using data from the 1000 Genomes database, we analysed whether the prevalence of alleles of the studied polymorphisms in the study group differed significantly compared to healthy individuals consisted of Europeans. No significant differences in allele distributions were found for both variants (*p* > 0.05). In the case of rs778986, the frequency of allele A was 16.7% in the study group and 18.0% in the reference population, respectively. Similarly, alleles A and T for rs3894326 also showed almost identical distributions in both compared groups. The obtained χ^2^ test values (rs778986: χ^2^ = 0.033; rs3894326: χ^2^ = 0.000) confirm the absence of statistically significant differences in allele frequencies between the study group and the reference population. These results suggest that the study group does not differ from the representative global population in terms of the analysed variants. The full results are presented in Table 2.

### 3.3. Analysis of SCFA Concentrations and Percentages in Patients

In the tested group, the highest average concentration was found for acetic acid (C2), with an average level of 38.45 nM/mg. The lowest values were recorded for caproic acid (C6) (average 3.90 nM/mg). The sum of SCFA in the samples averaged 96.82 nM/mg. In most of the analysed compounds (C3, C4, C5, C6, and the sum of SCFA), the normality test showed significant deviations from the normal distribution (*p* < 0.001), indicating asymmetry of distributions and the presence of extreme values. Only for acetic acid (C2) did the test show no significant deviation (*p* = 0.543). A similar trend was observed for the percentage content of SCFA, where the values for C3 often deviated from normality (*p* = 0.0001), while the C2, C4, C5, and C6 acids had distributions closer to normal (*p* > 0.05). Acetic acid accounted for the highest percentage of the SCFA pool (41.07%), while caproic acid accounted for the lowest (4.22%). The width of the confidence intervals (2.5–97.5 percentile) indicates high inter-individual variability, especially for butyric acid (C4) and the total sum of SCFA. The results of the analysis of SCFA concentrations and percentages are presented in Table 3.

The molar ratio of acetic acid:propionic acid:butyric acid = 3:1:1 was adopted as the reference (healthy) value [22]. In order to assess whether the study group met these criteria, individual molar ratios were calculated for each participant. The analysis showed that in 42 individuals (89%), this ratio deviated from the reference values, indicating a significant frequency of SCFA proportion disorders. Only five participants (11%) had a profile close to the norm. The most common deviation was an increase in acetic acid with a simultaneous decrease in propionate and/or butyrate, which is typical of reduced microbiota diversity. The full results of the analysis for all participants are presented in Table S1 in the Supplementary Materials.

### 3.4. Analysis of the Relationship Between the rs778986 Polymorphism of the *FUT3* Gene and the Concentration and Percentage of SCFA in the Study Group

**Figure 2:**
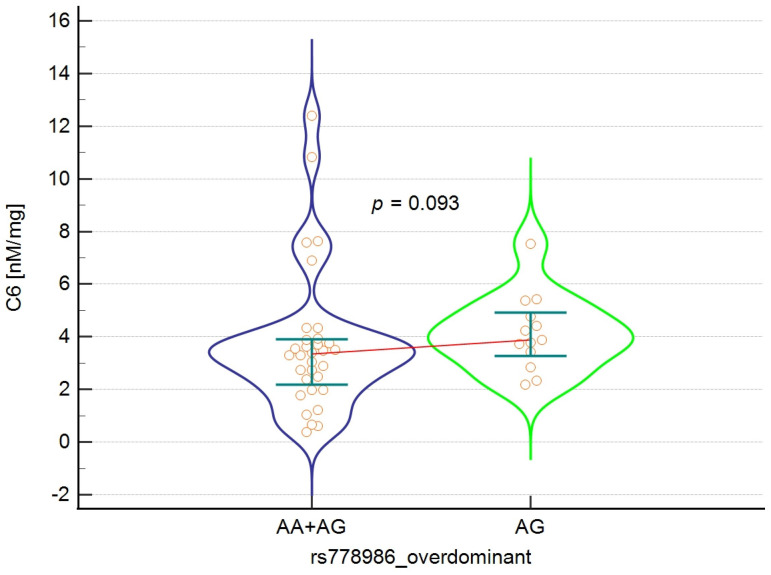
Violin plot illustrating the relationship between rs778986 polymorphism genotypes and C6 caproic acid concentration in an overdominant model. Error bars indicate interquartile ranges, the red line connects the medians, and the orange circles represent the individual results.

In the next stage of the study, the relationship between SCFA secretion and the rs778986 polymorphism genotype was examined in all inheritance models. Due to the low frequency of AA homozygotes, analysis in the recessive and codominant models would be subject to too high a risk of type I error. For this reason, we limited ourselves to dominant and overdominant models, which ensure the greater stability of estimation and statistical test power. In both models, high individual variability in SCFA concentrations was observed, as reflected in the wide interquartile ranges. This indicates the strong influence of environmental and microbiological factors, which may mask the potential genetic effect. Although statistical significance was not achieved (*p* = 0.093), there is a clear trend suggesting higher concentrations of caproic acid (C6) in AG heterozygotes in the overdominant model. Figure 2 shows the characteristic shape of the distribution in the AG group, indicating the presence of several higher values that may account for the observed trend. It is worth noting that despite the increased dispersion, the medians remain similar between groups. Detailed results are presented in Table 4 and Table 5 and Figure 2.

### 3.5. Analysis of the Relationship Between the rs3894326 Polymorphism of the *FUT3* Gene and the Concentration and Percentage of SCFA in the Study Group

**Figure 3:**
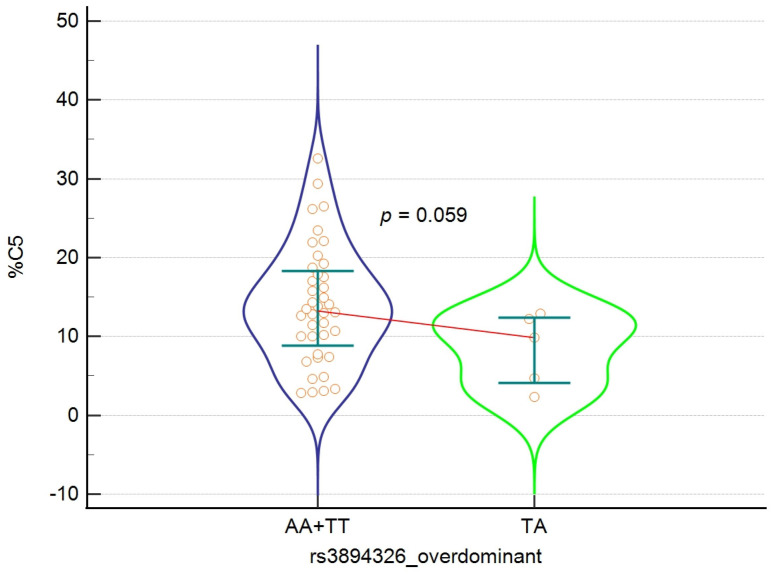
Violin plot illustrating the relationship between rs3894326 polymorphism genotypes and the percentage of C5 valeric acid in the overdominant model. Error bars indicate interquartile ranges, the red line connects the medians, and the orange circles represent individual results.

The final stage of the study was to check how SCFA concentration changes depending on the rs3894326 polymorphism genotype in the analysed inheritance models. Due to the low frequency of TT homozygotes, analyses of the recessive and codominant models were omitted and only recessive and overdominant models, which ensure the greater stability of estimation and statistical test power, were taken into account. No significant difference in SCFA concentrations with respect to genotypes in the analysed inheritance models was found. The distributions were broad and showed significant individual heterogeneity, suggesting the strong influence of environmental factors (including diet, lifestyle, and microbiota composition) that may mask the potential influence of the polymorphism itself. The results are presented in Table 6 and Table 7. In the overdominant model, statistical significance (*p* = 0.059) was observed for the percentage of valeric acid (C5), which was lower in TA heterozygotes compared to the combined AA+TT homozygote group. Figure 3 shows clear differences in the shape of the distributions between groups, especially greater variability and the presence of extreme values in the homozygous group (AA+TT). This may suggest that the effect of rs3894326 on C5 metabolism, if any, is non-linear and manifests itself mainly under host- or microbiota-specific conditions. Detailed results are presented in Table 7 and Figure 3.

Importantly, the present study (n ≈ 45–46 for genotyped samples) was not designed to demonstrate equivalence (i.e., “no effect”), and several genotype categories were rare (e.g., rs778986 AA and rs3894326 TT), which limits statistical power—particularly for codominant and recessive models. Therefore, non-significant p-values should be interpreted as insufficient evidence of association rather than definitive proof of absence of effect. The observed trend-level signals (rs778986 and C6, *p* = 0.093; rs3894326 and %C5, *p* = 0.059) further suggest that modest genotype-related differences cannot be excluded and require replication in larger cohorts.

While post hoc power calculations are sometimes requested, they can be misleading in the context of sparse cells and highly unbalanced subgroup sizes; therefore, we emphasise cautious interpretation of non-significant results and the need for adequately powered replication studies.

## 4. Discussion

Numerous microorganisms, referred to as gut microbiota, inhabit the human digestive tract. They are important for the body, as they participate in maintaining both immune and metabolic homeostasis. Several diseases have been identified so far that are accompanied by specific changes in the composition of the microbiota, but no clear cause-and-effect mechanisms leading to their development have been established [23].

One of the most important bacterial metabolites are SCFAs, which are produced as a result of the fermentation of complex carbohydrates. It is now known that SCFAs are essential for the proper functioning of the body [24]. Furthermore, these compounds play a key role in regulating the gut–brain axis through their ability to modulate the activity of the neuroendocrine system. It is worth noting that in the course of intestinal diseases accompanied by increased blood–brain barrier permeability, the translocation of bacterial products may intensify the secretion of pro-inflammatory cytokines, which exacerbates the inflammatory process [25]. SCFAs also participate in the regulation of lipid metabolism homeostasis, the deficiency of which may affect the integrity of the myelin sheath, neuroprotective mechanisms, microglial maturation, and CNS repair and remyelination processes [26]. Systemic injuries leading to a massive immune response can induce inflammation in the CNS through microglia activation, increased cytokine secretion and lymphocyte influx into the brain, resulting in a pro-inflammatory environment in the brain that negatively affects neuronal function [27,28]. Direct connections through branches of the sympathetic nervous system enable the CNS to be involved in maintaining intestinal homeostasis and immune regulation, and also allow for microbiota metabolites to influence CNS function [23,29,30].

An important element in the pathogenesis of MS—a chronic inflammatory disease of the central nervous system leading to the degradation of myelin sheaths—is an imbalance between pro- and anti-inflammatory cells. The course of MS is varied and initially characterised by reversible episodes of neurological deficits, which, over time, progress to a gradual and progressive deterioration of neurological function [31]. The gut microbiota is particularly important in this context, as dysbiosis is often observed in patients with MS [32]. Given the ability of SCFAs—especially butyrate—to induce Treg polarisation, targeted modulation of the microbiota composition towards beneficial metabolites may represent a potential therapeutic approach. Studies conducted by Haghikia et al. showed that administration of SCFA to mice with experimental autoimmune encephalomyelitis (EAE) resulted in improved clinical status [33]. Furthermore, butyrate treatment limited the demyelination process and supported remyelination by inducing the maturation and differentiation of oligodendrocytes. Acetate supplementation, on the other hand, led to increased acetyl-CoA metabolism and histone acetylation, which contributed to the protection of lipid resources in the spinal cord and partial prevention of EAE symptoms [34,35]. On this basis, it can be hypothesised that increased SCFA production in MS patients may correlate with a milder course of the disease.

In addition, researchers are interested in the *FUT3* gene, whose expression may influence the composition of the gut microbiota by participating in the biosynthesis of ABH and Lewis blood group antigens. These antigens serve as binding sites for specific microorganisms, such as noroviruses, rotaviruses, *Helicobacter pylori*, and *Campylobacter jejuni*. The expression of human blood group antigens (HGBA) in the intestine, regulated by the *FUT2* and *FUT3* genes, modifies the microbiota by adding fucose residues to precursor substrates. Dysbiosis, resulting from different *FUT3* gene activity, can lead to disturbances in SCFA secretion and thus to disruption of communication between the microbiota and the immune system [36].

Based on current data, the aim of this study was to assess the impact of rs778986 and rs3894326 polymorphisms of the *FUT3* gene on SCFA secretion in patients with MS from the West Pomeranian region. The analysis included 47 patients with a clinical diagnosis of MS who were under the care of the Provincial Centre for Demyelinating Diseases in Szczecin. The frequency of the studied genotypes in the group of patients and healthy individuals was compared, and the concentrations and percentage content of SCFA in faeces were assessed in relation to the presence of the studied polymorphisms.

The results of our study show that the distribution of genotypes of rs778986 and rs3894326 polymorphisms of the *FUT3* gene was consistent with the Hardy–Weinberg equilibrium and did not differ significantly from the control population, as confirmed by data from the European population from the 1000 Genomes database shown in Table 2. The high concordance of allele frequencies between groups indicates no systematic deviations and confirms the reliability of the genotypic data.

The average concentrations for the three main SCFAs shown in Table 3 were as follows: acetic acid—38.5 nM/mg, propionic acid—14.05 nM/mg, and butyric acid—28.15 nM/mg. In the case of these values, the normal molar ratio (3:1:1) confirmed repeatedly by studies [37,38,39,40], was disturbed, with deviations from the reference affecting 89% of patients. The highest concentration was recorded for acetic acid and the lowest for caproic acid (3.90 nM/mg). The literature shows that SCFA concentrations in MS patients differ from those observed in healthy individuals. Kim et al. and Simon et al. confirmed lower acetic acid concentrations in MS patients [41,42], while Zeng et al. also observed reduced levels of butyrate and propionate [43]. In a study by Saresella et al., SCFA concentrations were measured in the serum of patients with MS and healthy controls. They found that serum caproic acid levels were significantly higher in people with MS compared to the control group, while concentrations of anti-inflammatory butyric acid were lowered. Higher caproic acid levels correlated with an increased number of pro-inflammatory lymphocytes in peripheral blood, while a higher butyric acid/caproic acid ratio correlated with a higher number of Tregs. This condition may reflect intestinal microbiota dysbiosis and a disrupted intestinal barrier, which could contribute to chronic inflammation in people with MS [44]. Imbalances in SCFA molar ratios are often interpreted as a marker of intestinal dysbiosis, especially when reduced levels of butyrate are observed—a key metabolite with anti-inflammatory and trophic effects on colonocytes. An excess of acetate relative to other SCFAs may indicate a predominance of fibre-fermenting bacteria towards C2-producing pathways. Such a high frequency of abnormal ratios may result from dietary differences (low fibre intake, high simple sugar content), the use of drugs affecting the microbiota, or diverse intestinal bacterial composition.

The analysis did not reveal any statistically significant effect of rs778986 and rs3894326 polymorphisms on SCFA secretion in any of the inheritance models considered, as shown in Table 4, Table 5, Table 6 and Table 7. This result suggests that the analysed *FUT3* gene variants are unlikely to play a key role in the regulation of SCFA metabolism in the studied population. However, it should be emphasised that the lack of statistical significance in the observed correlations does not constitute clear evidence of the absence of a biological effect. It is possible that there is a subtle, real impact of the analysed polymorphisms, which has remained undetected due to limited statistical power resulting from the relatively small sample size. Only a tendency towards higher concentrations of caproic acid (C6) was observed in heterozygotes in the case of rs778986, as shown in Table 5. This may indicate a potential heterozygous advantage effect, whereby having two different alleles may modify the metabolic activity of the microbiota or influence the modulation of the host’s immune response, which in turn affects the SCFA profile. These results provide a basis for further exploration of the relationship between host genetic variability and intestinal bacterial metabolism. A tendency towards increased percentage content of valeric acid (C5) was also observed in homozygotes for rs3894326, as shown in Table 7. This may indicate a possible heterozygous advantage towards reduced C5 production, although, due to the very small size of the TA group, these conclusions should be treated as preliminary. These results suggest a possible contribution of polymorphisms to the modulation of SCFA metabolism.

Caproic acid, a six-carbon fatty acid naturally present in animal fats, can also be produced by intestinal microorganisms, including bacteria of the genus Prevotella [35,36]. It exhibits bactericidal properties and participates in the differentiation of Th1 and Th17 cells, which play an important role in the pathogenesis of MS [33]. Research by Saresella et al. showed higher concentrations of caproic acid in MS patients compared to healthy individuals, which correlated with an increased number of bacteria capable of producing it [44]. Valeric acid, in turn, participates in the processes of self-renewal and differentiation of intestinal stem cells (ISCs), regulated by complex signalling mechanisms [45,46]. Zhu et al. demonstrated that this acid promotes ISC renewal by inducing the expression of serotonin 5-HT receptors in intestinal neurons. Activation of these receptors stimulates the production of prostaglandin E2 in macrophages, which promotes ISC regeneration [47]. Changes in the composition of the microbiota caused by *FUT3* gene polymorphisms, including a reduction in the population of SCFA-producing bacteria, may result in reduced SCFA secretion, leading to the decreased bactericidal activity of caproic acid, limited differentiation of Th1 and Th17 cells, and weakened self-renewal and differentiation processes of intestinal stem cells supported by valeric acid, consequently modulating the immune response relevant to the pathophysiology of multiple sclerosis [11,48,49,50].

Our study has an important limitation, being low statistical power for genotype–phenotype analyses. Although the overall sample size was adequate for descriptive profiling, genotype subgroup sizes were small and, for some variants, certain homozygous genotypes were rare. This imbalance reduces power, inflates uncertainty, and limits the interpretability of “null” findings—particularly under codominant or recessive models. For this reason, we focused on dominant/overdominant contrasts; however, even under these models, the study may only detect moderate-to-large differences in SCFA levels. Consequently, the absence of statistically significant associations in some comparisons should be interpreted as insufficient evidence of association rather than conclusive evidence of no effect. Replication in larger, well-powered cohorts (ideally enabling model-robust analyses and adjustment for multiple testing) is required to determine whether smaller genotype-related effects exist. Only the replication of these results in independent cohorts and their confirmation in studies with sufficiently high power will allow for a more unambiguous assessment of the role of the polymorphisms role in the secretion of SCFA in MS. The other limitation includes the lack of dietary assessment, a factor which might influence SCFA synthesis to a large extent. However, as stated in the recent study the association/causation is under the debate [51]. Full demographic and clinical data were not extensively collected by our team. Future studies that include such variables (especially treatment), which might affect the composition of the gut microbiota and, consequently, SCFA levels, are warranted [52].

In the absence of effective causal therapy for MS, new therapeutic targets are constantly being sought. SCFAs, produced by gut bacteria, are of fundamental importance in regulating the immune response, including by modulating the secretion of cytokines that are overly active in MS. In this context, particular attention is paid to factors that may influence SCFA production, including genetic determinants such as genes encoding fucosyltransferases. Although the presented study indicates that the analysed *FUT3* gene polymorphisms do not have a significant impact on SCFA synthesis, as a pioneering study, it opens up new perspectives for research into the genetic determinants of lipid metabolism by the microbiota. Further, more extensive analyses are needed, covering both host and microorganism genetics, which may in the future enable the development of innovative and safe therapeutic interventions targeting the SCFA metabolism.

## 5. Conclusions

In summary, the analyses indicate that the rs778986 and rs3894326 polymorphisms of the *FUT3* gene are not overrepresented among MS patients, and their frequency does not differ significantly from that in the healthy European population from the 1000 Genomes database. The observed influence of genetic variants on valeric and caproic acid secretion suggests that the *FUT3* gene may play a modulating but minor role in the metabolism of certain SCFA. However, the lack of effect on overall SCFA concentrations means that these polymorphisms are unlikely to be key biomarkers or therapeutic targets in MS at the current stage of knowledge. Since overall SCFA concentrations are disrupted in MS patients but are not directly attributable to *FUT3* polymorphisms, the clinical focus should be directed more towards other factors (diet, probiotics). The results support the thesis that MS patients have intestinal dysbiosis, which may influence disease progression through SCFA alterations, even if not dependent on *FUT3*. These findings will help refine future research on microbiome by indicating that host glycosylation genes such as *FUT3* may exert only subtle, pathway-specific effects on microbial metabolite profiles, thereby encouraging broader, integrative analyses that combine host genetics, microbiome composition and function, and environmental factors. Clinically, this suggests that therapeutic strategies in MS are more likely to benefit from targeting modifiable microbiome-related factors (e.g., diet or probiotics) rather than single host genetic variants. The results obtained emphasise the importance of further research into the genetic and microbiological factors regulating SCFA metabolism, which may provide a basis for the development of new therapeutic strategies in MS.

## Figures and Tables

**Table 1:** Distribution of genotypes in the studied inheritance models.

Gene Polymorphism	Inheritance Model	Genotype	n	%
*FUT3* rs778986 n = 45	Codominant	AA	1	2.2%
		AG	13	28.9%
		GG	31	68.9%
	Dominant	GG	31	68.9%
		AA+AG	14	31.1%
	Overdominant	AA+GG	32	71.1%
		AG	13	28.9%
	Recessive	AA	1	2.2%
		AG+GG	44	97.8%
*FUT3* rs3894326 n = 46	Codominant	AA	40	87.0%
		AT	5	10.9%
		TT	1	2.1%
	Dominant	TT	1	2.1%
		AA+AT	45	97.9%
	Overdominant	AA+TT	41	89.1%
		AT	5	10.9%
	Recessive	AA	40	87.0%
		AT+TT	6	13.0%

n—number.

**Table 2:** Comparison of allele frequencies of studied *FUT3* polymorphisms between the study group and the 1000 Genomes reference population.

***FUT3* rs778986**	**A (%)**	**G (%)**	**P**	**χ^2^**
Study group	15 (16.7%)	75 (83.3%)	0.857	0.033
The 1000 Genomes Project	362 (18.0%)	1650 (82.0%)		
***FUT3* rs3894326**	**A (%)**	**T (%)**	**P**	**χ^2^**
Study group	85 (92.4%)	7 (7.6%)	1.000	0.000
The 1000 Genomes Project	1851 (92.0%)	161 (8.0%)		

P—statistical significance, χ^2^—chi-square.

**Table 3:** Concentrations and percentage share of SCFA in the study group.

**nM/mg**	**N**	**Min**	**Max**	**M**	**Me**	**SD**	**2.5–97.5 P**
Acetic acid C2	47	5.69	76.47	38.45	38.60	15.47	10.49–72.24
Propionic acid C3	47	3.73	46.03	14.05	12.69	9.03	3.92–44.38
Butyric acid C4	47	5.03	113.61	28.15	22.25	22.25	5.72–108.25
Valeric acid C5	47	2.31	44.65	12.28	9.94	8.33	2.44–34.66
Caproic acid C6	47	0.37	12.41	3.90	3.49	2.47	0.53–11.34
Total SCFA in samples	47	31.61	260.84	96.82	90.88	44.66	33.09–247.74
**%**	**N**	**Min**	**Max**	**M**	**Me**	**SD**	**2.5–97.5 P**
Acetic acid C2	47	6.41	65.51	41.07	41.60	9.81	19.74–60.46
Propionic acid C3	47	5.18	37.97	14.46	14.41	5.50	6.52–30.39
Butyric acid C4	47	9.13	51.47	26.95	25.07	9.51	11.08–48.49
Valeric acid C5	47	2.32	32.56	13.31	12.91	7.26	2.70–30.39
Caproic acid C6	47	0.52	10.09	4.22	3.91	2.14	0.75–9.72

N—number of subjects; Min—minimum; Max—maximum; M—mean; Me—median; SD—standard deviation; 2.5—97.5 P—interquartile range.

**Table 4:** Analysis of the relationship between genotypes of the rs778986 polymorphism of the *FUT3* gene and the concentration and percentage of SCFA in the dominant model.

**[nM/mg]**	**Dominant** **rs778986 = “GG”**			**Dominant** **rs778986 = “AA+AG”**			**P**
	**N**	**Me**	**IQR**	**N**	**Me**	**IQR**	
Acetic acid C2	31	39.91	13.07–68.31	14	38.56	5.69–76.47	0.845
Propionic acid C3	31	11.42	3.81–42.30	14	12.84	5.91–46.03	0.980
Butyric acid C4	31	21.91	5.31–100.31	14	23.32	7.76–105.6	0.641
Valeric acid C5	31	9.29	2.36–39.29	14	13.03	5.1–29.85	0.186
Caproic acid C6	31	3.41	0.44–11.97	14	3.83	2.18–7.54	0.170
Total SCFA in samples	31	92.58	32.21–222.60	14	88.98	39.49–260.84	0.941
**%**	**Dominant** **rs778986 = “GG”**			**Dominant** **rs778986 = “AA+AG”**			**P**
	**N**	**Me**	**IQR**	**N**	**Me**	**IQR**	
Acetic acid C2	31	41.60	26.96–63.45	14	41.88	6.41–55.46	0.902
Propionic acid C3	31	13.30	5.72–34.89	14	14.69	9.11–17.65	0.825
Butyric acid C4	31	25.16	9.92–50.26	14	25.03	19.56–46.64	0.864
Valeric acid C5	31	11.71	2.47–31.67	14	13.52	4.89–26.18	0.281
Caproic acid C6	31	3.75	0.61–9.40	14	4.337	1.07- 9.54	0.281

N—number of subjects; Me—median; P—statistical significance; IQR—interquartile range.

**Table 5:** Analysis of the relationship between genotypes of the rs778986 polymorphism of the *FUT3* gene and the concentration and percentage of SCFA in the overdominant model.

**[nM/mg]**	**Overdominant** **rs778986 = “AG”**			**Overdominant** **rs778986 = “AA+GG”**			**P**
	**N**	**Me**	**IQR**	**N**	**Me**	**IQR**	
Acetic acid C2	13	38.60	5.69–76.47	32	39.02	13.09–68.13	1
Propionic acid C3	13	12.87	5.91–46.03	32	11.32	3.82–42.19	0.841
Butyric acid C4	13	24.35	7.76–105.66	32	21.58	5.34–99.10	0.499
Valeric acid C5	13	13.08	5.11–29.85	32	9.40	2.36–38.80	0.193
Caproic acid C6	13	3.88	2.18–7.54	32	3.35	0.44–11.93	0.093
Total SCFA in samples	13	89.24	39.49–260.84	32	91.73	32.26–220.89	0.764
**%**	**Overdominant** **rs778986 = “AG”**			**Overdominant** **rs778986 = “AA+GG”**			**P**
	**N**	**Me**	**IQR**	**N**	**Me**	**IQR**	
Acetic acid C2	13	40.87	6.41–55.46	32	41.62	27.03–63.26	0.764
Propionic acid C3	13	14.97	9.11–17.65	32	12.99	5.77–34.61	0.707
Butyric acid C4	13	24.98	19.56–46.64	32	25.36	10.00–50.15	0.900
Valeric acid C5	13	12.95	4.89–26.18	32	12.18	2.49–31.59	0.341
Caproic acid C6	13	4.53	1.09–9.54	32	3.70	0.62–9.34	0.211

N—number of subjects; Me—median; P—statistical significance; IQR—interquartile range.

**Table 6:** Analysis of the relationship between genotypes of the rs3894326 polymorphism of the *FUT3* gene and the concentration and percentage of SCFA in the recessive model.

**[nM/mg]**	**Recessive** **rs3894326 = “AA”**			**Recessive** **rs3894326 = “AA”**			**P**
	**N**	**Me**	**IQR**	**N**	**Me**	**IQR**	
Acetic acid C2	40	38.33	9.25–71.37	6	39.41	24.06–63.30	0.7941
Propionic acid C3	40	11.32	3.87–44.81	6	12.78	5.57–16.87	0.625
Butyric acid C4	40	22.08	5.54–84.97	6	23.00	13.80–65.24	0.493
Valeric acid C5	40	10.01	2.52–37.25	6	9.01	2.50–17.09	0.514
Caproic acid C6	40	3.524	0.49–9.60	6	3.11	1.22–12.41	0.794
Total SCFA in samples	40	90.80	32.70–210.76	6	87.01	62.60–172.95	0.922
**%**	**Recessive** **rs3894326 = “AA”**			**Recessive** **rs3894326 = “AA”**			**P**
	**N**	**Me**	**IQR**	**N**	**Me**	**IQR**	
Acetic acid C2	40	41.74	16.29–61.77	6	39.49	33.05–50.01	0.819
Propionic acid C3	40	14.29	7.17–32.36	6	12.46	5.18–20.27	0.625
Butyric acid C4	40	24.99	10.57–46.53	6	28.09	21.73–51.47	0.282
Valeric acid C5	40	13.27	2.89–30.95	6	11.05	2.32–22.09	0.240
Caproic acid C6	40	4.03	0.69–9.82	6	3.41	1.32–7.17	0.602

N—number of subjects; Me—median; P—statistical significance; IQR—interquartile range.

**Table 7:** Analysis of the relationship between genotypes of the rs3894326 polymorphism of the *FUT3* gene and the concentration and percentage of SCFA in the overdominant model.

**[nM/mg]**	**Overdominant** **rs3894326 = “TA”**			**Overdominant** **rs3894326 = “AA+TT”**			**P**
	**N**	**Me**	**IQR**	**N**	**Me**	**IQR**	
Acetic acid C2	5	40.22	26.04–63.30	41	38.14	9.42–71.11	0.407
Propionic acid C3	5	12.87	5.57–16.87	41	11.23	3.88–44.75	0.986
Butyric acid C4	5	23.85	13.80–65.24	41	22.14	5.56–83.94	0.448
Valeric acid C5	5	8.08	2.50–17.09	41	10.30	2.54–36.88	0.282
Caproic acid C6	5	2.33	1.22–12.41	41	3.56	0.50–9.54	0.584
Total SCFA in samples	5	92.58	62.60–172.95	41	90.72	32.76–208.26	0.659
**%**	**Overdominant** **rs3894326 = “TA”**			**Overdominant** **rs3894326 = “AA+TT”**			**P**
	**N**	**Me**	**IQR**	**N**	**Me**	**IQR**	
Acetic acid C2	5	41.60	36.60–50.01	41	41.63	16.78–61.58	0.764
Propionic acid C3	5	15.80	5.18–20.27	41	14.17	7.17–32.08	0.958
Butyric acid C4	5	25.76	21.73–51.47	41	24.99	10.64–46.52	0.448
Valeric acid C5	5	9.88	2.32–12.91	41	13.48	2.89–30.87	0.059
Caproic acid C6	5	3.17	1.32–7.17	41	4.14	0.70–9.80	0.315

N—number of subjects; Me—median; P—statistical significance; IQR—interquartile range.

## Data Availability

The raw data supporting the conclusions of this article will be made available by the authors on request.

## Supplementary Materials

The following supporting information can be downloaded at https://www.mdpi.com/article/10.3390/nu18010062/s1. Table S1: Concentrations of individual SCFAs and their ratio in each patient in the study group.
