# Supplementary material for: The Relationship Between Short-Chain Fatty Acid Secretion and Polymorphisms rs3894326 and rs778986 of the FUT3 Gene in Patients with Multiple Sclerosis—An Exploratory Analysis
Source: Nutrients. 2025 Dec 24;18(1):62. doi: 10.3390/nu18010062 (PMC12787777; doi:10.3390/nu18010062)
Supplement: Supplementary file 1 [file nutrients-18-00062-s001.zip › nutrients-4034663-supplementary.pdf]

Table S1. Concentrations of individual SCFAs and their ratio in each patient in the study group.

Patient	Acetate [nM/mg]	Propionate [nM/mg]	Butyrate [nM/mg]	Ratio		
				3	1	1
1	4,0216	0,5568	5,5375	8	1	11
2	4,814	1,7251	4,3573	3	1	3
3	3,8141	1,3619	2,7471	3	1	2
4	3,8514	0,9254	2,2294	4	1	2
5	6,6259	1,6638	2,8417	4	2	2
6	4,3659	1,895	2,8073	3	1	2
7	3,2917	0,9178	1,8968	3	1	2
8	4,3024	2,039	6,4281	2	1	3
9	3,0019	0,8293	2,6396	3	1	2
10	4,6304	1,6873	2,385	3	1	2
11	1,8562	0,8436	1,1327	2	1	1
12	3,3872	1,2951	2,4418	3	1	2
13	0,5689	1,4988	4,1372	1	3	8
14	4,1764	1,631	2,5621	3	1	2
15	6,1883	1,2801	2,182	4	1	2
16	5,0543	4,3589	1,379	4	3	1
17	2,4059	0,6631	2,214	4	1	3
18	2,6043	1,2689	1,3804	2	1	1
19	1,3759	0,4011	0,5033	3	1	1
20	4,3194	0,6764	3,7138	6	1	5
21	4,075	1,4646	2,4349	3	1	2
22	4,487	1,005	1,5274	4	1	2
23	4,0806	0,9823	3,4278	4	1	3
24	1,6277	1,0908	2,1905	2	1	2
25	4,4246	1,7522	3,1639	3	1	2
26	3,9906	1,09	2,3772	4	1	2
27	3,4766	0,6575	2,0538	5	1	3
28	1,9178	0,5603	0,6044	4	1	1
29	3,7961	0,8972	2,0487	4	1	2

30	7,6474	4,6025	10,5662	2	1	3
31	3,1727	1,1226	2,8473	3	1	2
32	4,155	1,5787	0,861	5	2	1
33	3,8598	1,2869	1,7694	3	1	1
34	4,6042	1,691	2,2245	3	1	1
35	6,2796	2,0287	0,8753	6	2	1
36	2,485	0,6748	1,8247	4	1	3
37	3,7831	1,1418	1,8296	3	1	2
38	4,8672	1,7562	3,2518	3	1	2
39	2,7577	0,571	2,1257	5	1	4
40	1,2802	0,3732	0,8088	3	1	2
41	1,6936	0,5911	0,7761	3	1	1
42	2,6635	0,9952	2,1195	2	1	2
43	3,3876	1,883	1,1622	3	2	1
44	6,3295	1,4915	6,5239	4	1	4
45	2,8871	0,9589	0,929	3	1	1
46	7,0206	3,8908	11,3613	2	1	3
47	5,3156	2,3542	5,0897	2	1	2
